# Activation of chicken macrophages by isomalto/malto-polysaccharide (IMMP) is facilitated by toll-like receptor 4 (TLR4)

**DOI:** 10.1016/j.psj.2025.105690

**Published:** 2025-08-14

**Authors:** Adil Ijaz, Max Putman, Femke Broere, Victor P.M.G. Rutten, Christine A. Jansen, Edwin J.A. Veldhuizen

**Affiliations:** a Division Infectious Diseases and Immunology, Department Biomolecular Health Sciences, Faculty of Veterinary Medicine, Utrecht University, Utrecht, the Netherlands; b Department of Veterinary Tropical Diseases, Faculty of Veterinary Science, University of Pretoria, Pretoria, South Africa; c Cell Biology and Immunology group, Department of Animal Sciences, Wageningen University & Research, Wageningen, the Netherlands

**Keywords:** IMMP, TLR4, HD11 cells, Chicken, Macrophage

## Abstract

Isomalto/malto-polysaccharide (IMMP) is a digestion resistant starch that has shown to increase SCFA production in the gut microbiota of human and mice to promote intestinal health. Like other prebiotics, it escapes enzymatic digestion in the small intestine and reaches the colon intact where it can interact with host epithelial and immune cells. Recently it was shown that *in vitro* IMMP can bind TLR4 on human macrophages resulting in their activation. The purpose of the present study was to investigate putative immune activation effects of IMMP upon direct interaction with chicken macrophages. IMMP stimulation *in vitro* caused morphological changes in the macrophage cell line HD11 reflected as reduction in cell size and numbers of cytoplasmic projections. Using the extracellular TLR4 blocking agent LPS-RS, it was shown that IMMP stimulation of HD11 cells involved TLR4 signaling. In addition, it activated HD11 cells and monocyte derived macrophages, resulting in the production of nitric oxide and increased phagocytic activity of HD11 cells. In conclusion, our results demonstrate an *in vitro* effect of IMMP on chicken macrophages which suggests that this carbohydrate compound may have immunomodulatory potential, hence may be used in the future as an alternative strategy to strengthen immune responsiveness to control intestinal infections.

## Introduction

Prebiotics are commonly used as functional foods, both in animals and humans, which are selectively fermented in the colon to confer health benefits. Prebiotics alter the function or composition of the gut microbiota (Gibson, et al., 2010), and also exert microbiota-independent effects through direct interaction with intestinal epithelial and resident innate immune cells (Pujari and Banerjee, 2021). Many commonly used food items, such as garlic, chicory, honey, and wheat have prebiotic properties. Moreover, prebiotic compounds such as galacto-oligosaccharide (GOS) are also manufactured on an industrial scale using starch or lactose as raw material (Al-Sheraji, et al., 2013), highlighting the market potential of these compounds as relatively cheap feed additives.

The main mechanism by which prebiotics contribute to health benefits is their selective use as energy source for beneficial gut microbiota such as *Lactobacilli* and *Bifidobacteria* (Markowiak and Śliżewska, 2017), which leads to higher quantities of short chain fatty acids (SCFA) as fermentation products (Gibson, et al., 2017; Gibson, et al., 2004). SCFA lower the pH of the gut which promotes growth of *Firmicutes* (Walker, et al., 2005), but it also nourishes intestinal cells to maintain or even strengthen the gut barrier integrity (Klatt, et al., 2013). Due to their health promoting effects, prebiotics may be used for the treatment of intestinal inflammatory disorders, such as IBD, to restore gut homeostasis and subsequently gut health (Ewaschuk and Dieleman, 2006). These characteristics of prebiotics can therefore reduce the incidence of intestinal infections, thus lowering the use of antibiotics to treat infections (Wilson and Whelan, 2017).

Isomalto/malto-polysaccharides (IMMPs) are widely used as dietary fiber because of their prebiotic potential (Leemhuis, et al., 2014). IMMP is a water soluble polysaccharide that is produced by enzymatic modification of starch using the starch converting enzyme 4,6-α-glucanotransferase (GTFB) from *Lactobacillus reuteri* (van der Zaal, et al., 2018) and *Streptococcus thermophilus* (Li, et al., 2021). The GTFB enzyme acts as debranching enzyme that converts α-(1-4)- and introduces α-(1-6) glycosidic linkages throughout IMMP, thus making it resistant to degradation by α-amylases (Leemhuis, et al., 2014). Due to this, IMMPs escape digestion in the upper gastrointestinal tract and are available as energy source for microbiota in the large intestine (Leemhuis, et al., 2014).

The beneficial effects of IMMPs on the host’s gut health are multifaceted just as several other prebiotics. Several *in vivo* as well as *in vitro* studies have described possible mechanisms by which IMMPs may improve gut health. It has been demonstrated that *in vitro* fermentation of IMMPs results in production of the SCFAs such as acetic acid and propionic acid (Leemhuis, et al., 2014). Additionally, *in vitro* fermentation studies using fecal inoculum of humans as a microbial source have shown that IMMP fermentation significantly improved the relative abundance of *Lactobacillus* and *Bifidobacterium* as well as SCFAs, especially acetic acid and succinic acid (Gu, et al., 2018). SCFAs can be used locally by colonocytes to support intestinal barrier function and may also be absorbed across the intestinal epithelium into the bloodstream, and reach other organs and tissues (Tan, et al., 2014). This shows that IMMPs have a beneficial effect on gut microbiota by increasing the relative abundance of gut beneficial microbes as well as by increasing the production of SCFAs. In addition to improving beneficial gut microbiota and production of SCFAs, IMMPs may also improve gut health by modulation of host immune functions as they may interact with intestinal immune cells during the retention time in the gut. It has been shown, in humans, that IMMP attenuated the TNFα, 1L-1β, and IL-6 cytokine response of immature dendritic cells when exposed to fecal inoculum of 4 weeks old infants (Logtenberg, et al., 2021). The authors also showed that IMMP was actually not degraded by the fecal bacteria, indicating that IMMP may have exerted an immunomodulatory effect on immature DCs, but this was not further investigated in this study (Logtenberg, et al., 2021).

Since the emergence of antimicrobial resistance, many carbohydrate compounds (polysaccharides, oligosaccharides, α-glucans, and pectins) have been tested as an alternative for antibiotics in poultry farming (Andrew Selaledi, et al., 2020; La Ragione and Burton, 2023), and it is an ongoing area of research. Carbohydrates have been shown to promote gut health in poultry through promoting the prevalence of healthy gut microbiota (van den Broek, et al., 2008), increased villus height and surface area in the intestine (Hooge, et al., 2003), and immunomodulation (Zmrhal and Slama, 2020). Recently, we have shown that IMMP attenuates responsiveness of human THP1 cells through interaction with toll-like receptor (TLR) 2 and TLR4 (Silva-Lagos, et al., 2025). However, no information is available about immunomodulatory properties of IMMP on chicken immune cells in relation to TLRs. Considering the difference in human and chicken TLRs, especially TLR2 which has two isoforms (chTLR2 type 1 and 2) (Keestra, et al., 2013) unlike in humans, it is unclear if IMMP can exert a similar effect in chicken. The purpose of this study was to determine possible immunomodulatory properties of IMMP in direct interaction with chicken macrophages *in vitro*.

## Materials and methods

### Identification of human, mouse, and chicken TLR4 protein sequences

Amino acid sequences of chicken (FJ915519.1), human (NM_138554.5), and mouse (AF110133.1) full length TLR4 were obtained using the NCBI database https://www.ncbi.nlm.nih.gov/. To determine the identity matrix, protein sequences were aligned using ClustalW (Thompson, et al., 1994).

### Compounds and reagents

Isomalto/malto-polysaccharide-87 (IMMP), was provided by Royal Avebe, (Veendam, the Netherlands). It is obtained from potato starch and the process of generation and characterization of IMMP has been described before (Leemhuis, et al., 2014). The total α-(1-6) glycosyl content including both α-(1-4,6), and α-(1-6) glycosyl residues, monosaccharide composition, and molecular weight (10 kDa) has also been described (van der Zaal, et al., 2018). IMMP was dissolved at 10 mg/mL in either RPMI-1640 or DMEM media supplemented with 10% fetal calf serum (FCS) and 200 units/mL penicillin, 200 mg/mL streptomycin (P/S) (Gibco, Life Technologies Limited, Paisley, UK) and stored in -20°C for future use. TAK-242, an intracellular TLR4 antagonist, (also known as Resatorvid or CLI-095) was purchased from Selleckchem (S7455, Houston, USA). TAK-242 was dissolved in dimethyl sulfoxide (DMSO) at a stock concentration of 10 mM and stored at -20°C for future use. LPS-RS (Cat. Code tlrl-rslps), a surface TLR4 antagonist, was purchased from Invivogen (Toulouse, France) and dissolved in endotoxin free water at a stock concentration of 5 mg/mL and stored at -20°C for future use. LPS-RS is a lipopolysaccharide derived from *Rhodobacter sphaeroides*, that acts as a competitive antagonist of TLR4 by binding to TLR4/MD2 complex, thereby preventing downstream signaling.

### HD11 cell line

HD11, a chicken macrophage-like cell line (Beug, et al., 1979), was cultured in RPMI-1640 (Gibco, Life Technologies Limited, Paisley, UK) supplemented with 10% FCS, and P/S. The cells were passaged twice a week using 0.05% trypsin/EDTA (Gibco, Life Technologies Limited, Paisley, UK) upon reaching 90% confluency. HD11 cells were used between passage 2 to passage 15.

### Chicken blood monocyte derived macrophages (chicken MDMs)

Peripheral blood mononuclear cells (PBMCs) were isolated from blood of five 36 weeks-old healthy Novogen white layer chickens using a Ficoll density gradient as described earlier (He, et al., 2003), and stored at -140°C until further use. Monocytes were isolated from PBMCs and differentiated into macrophages as described earlier (Peng, et al., 2020). In short, PBMCs were seeded in a 96 wells plate, at 1 × 10^6^ cells per well, in 200 µL of RPMI-1640 supplemented with 10% FCS, and P/S. After 6 h of incubation at 41°C, supernatant was discarded, the attached cells were washed 3 times with PBS to remove all the non-adherent cells. Subsequently, the attached cells were maintained in RPMI media with FCS and P/S supplemented with recombinant granulocyte-macrophage colony-stimulating factor (GM-CSF) at a concentration of 1:1600 for 3 days at 41°C to differentiate monocytes to macrophages (Peng, et al., 2020). Recombinant GM-CSF was produced by transfecting COS-7 cells with pCI-neo (Promega Corporation, Madison, Wisconsin, USA) that expresses the relevant cytokine (kind gift from P. Kaiser and L. Rothwell, The Roslin Institute, Edinburgh, UK). The cells were then stimulated with 0.6-2.5 mg/mL IMMP, 50 ng/mL LPS-EB (Invivogen, Toulouse, France), or only media for 24 h. Each independent experiment was performed using a new PMBCs from the stored stock solutions.

### Alamar blue cell viability assay

The viability of HD11 and HEK-Blue cells after stimulation with IMMP was determined by the Alamar blue viability assay. HEK-Blue TLR4 and HD11 cells were stimulated with IMMP (0.6 to 2.5 mg/mL) or medium for 24 h. Cells treated with 1% Triton X-100 (Sigma-Aldrich, USA) served as negative (non-viable) control cells. After 24 h of stimulation, supernatants were removed and cells were incubated with 100 μL of 10% Alamar blue reagent (Thermo Fisher Scientific, USA) at 37°C, 5% CO_2_ for 4 h. Finally, the absorbance was measured at a wavelength of 570 nm with a reference at 600 nm, using a FLUOstar Omega microplate reader (BMG Labtech, Ortenberg, Germany).

### Nitric oxide production by macrophages measured by Griess assay

To determine the activation of HD11 cells and blood monocyte derived macrophages (MDMs), nitric oxide production was measured by the Griess assay (Hussain and Qureshi, 1997). For this, 50,000 HD11 cells or 100,000 chicken MDMs were stimulated with IMMP (0-2.5 mg/mL), LPS (100 ng/mL) for 24 h in a 96 well flat bottom plate. After stimulation, 50 µL of supernatant was collected in a new 96-well flat-bottom plate and then mixed with 50 µL of 10 mg/mL sulfanilamide (2.5% phosphoric acid) (Sigma, Missouri, USA) and 3 mg/ml N-(1-napthyl) ethylenediamine dihydrochloride (Sigma, Missouri, USA), respectively (Green, et al., 1982). The absorbance was measured at a wavelength of 550 nm using an iMark microplate absorbance reader (Bio-Rad Laboratories, Hercules, California, United States). The concentration of nitric oxide was determined using a standard curve of sodium nitrite having a concentration range from 0-100 µM.

### Phagocytosis of crimson beads by HD11 cells as assessed by flow cytometry

The phagocytic activity of HD11 cells after stimulation with IMMP was measured using the uptake of crimson beads (Invitrogen, Life Technologies Europe BV, Bleiswijk, The Netherlands). HD11 cells (2 × 10^5^) were seeded in a 24 well flat bottom plate. The next day, supernatant was removed and HD11 cells were stimulated with IMMP (0-2.5 mg/mL), LPS (100 ng/mL), and medium-only for 24 h at 37°C. Following stimulation, crimson beads were added to the HD11 cells at a 1:1 bead-to-cell ratio (2 × 10^5^ cells and 2 × 10^5^ beads). The HD11 cells were incubated for 4 h at 37°C, 5% CO_2_ to allow the cells to phagocytose the beads. Subsequently, the cells were harvested using Dulbecco’s phosphate-buffered saline (DPBS^-/-^; Lonza, Basel, Switzerland) supplemented with 0.25% trypsin/EDTA (Gibco, Life Technologies Limited, Paisley, UK) and centrifuged at 400 x g for 3 min. The cells were then transferred to a 96 well V-bottom plate, washed in DPBS^-/-^, and stained with 50 μL (1:400) of Zombie Aqua Fixable Viability Dye (BioLegend Inc., San Diego, CA, USA) for 20 min at 4°C in the dark. Finally, the cells were washed with 200 μL DPBS-/- followed by FACS buffer containing 0.5% bovine serum albumin (Sigma-Aldrich, Saint Louis, MO, USA) and 0.005% sodium azide (Sigma-Aldrich, Saint Louis, MO, USA) in DPBS^-/-^. The samples were resuspended in 200 μL of FACS buffer and analysed using the CytoFLEX LX flow cytometer and up to 50,000 cells in total were recorded. The fluorescence of HD11 cells was measured using a 660/10-nm wavelength bandpass filter after excitation with the 638-nm laser. The FACS data were analysed with FlowJo Software v. 10.8 and GraphPad Prism 9.

### Phagocytosis of crimson beads by HD11 cells as assessed by confocal microscopy

The uptake of crimson beads by HD11 cells after stimulation with IMMP was determined by confocal microscopy. For this, 2 × 10^5^ HD11 cells were seeded overnight at 37 °C on glass slides in a 24 well plate (Sigma-Aldrich, Merck, St. Luis, MO, USA). The next day, supernatant was removed and HD11 cells were stimulated with IMMP (2.5 mg/mL), LPS (50 ng/mL), and medium only for 24 h at 37 °C. After stimulation with IMMP, HD11 cells were incubated with crimson beads for 4 h at 37 °C. Following that, the supernatant was removed and HD11 cells were stained with the cell membrane marker WGA-AF488 (1:1000) for 2 h at RT. The cells were then fixed with 4% paraformaldehyde (PFA) (Alfa Aesar, Haverhill, MA, USA) for 30 min and permeabilized with 0.5% Triton X-100 solution for 10 min at RT. Nuclei were stained with 5 µg/mL DAPI for 5 min at RT. HD11 cells were washed twice for 5 min with washing buffer containing 0.05% Tween-20 and then mounted on Poly-L-lysine slides (Menzel Glazer GmbH & Co KG, Braunschweig, Germany). Samples were imaged using a Leica TCS SPEII microscope (Leica, Amsterdam, the Netherlands) and images were analysed using FIJI software (NIH. Version ImageJ 1.52r).

### Statistical analyses

Statistical analyses were performed using GraphPad Prism program version 9 (San Diego, CA, USA). The data are represented as mean ± SEM. Statistical significance was determined using one-way ANOVA with Dunnett’s multiple comparisons test, while *p* < 0.05 was considered as statistically significant.

## Results

### Comparison of human, mouse, and chicken TLR4 amino acid sequences

Amino acid sequence alignment of human, mouse, and chicken TLR4 was done for the whole protein sequence and showed that the total human and chicken TLR4 sequence are 46% identical (as shown in supplementary Fig. S1). The highest homology can be found in the intracellular (cytoplasmic) domain (ICD 57%) This domain largely exists of the TIR domain (60% homologous), which changes conformation upon LPS binding and initiates downstream signaling. The extracellular domain (ECD) that is involved in LPS binding has a much lower homology of only 32%. For comparison mouse and human sequences are 67%, 62%, 87% and 92% homologous for the full sequence, ECD, ICD and TIR, respectively.

### IMMP did not affect the viability of HD11

**Fig. 1:**
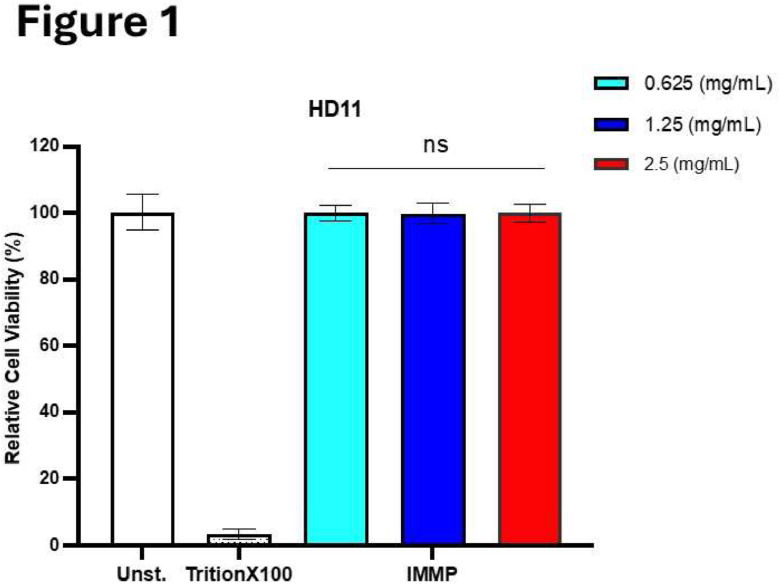
IMMP stimulation did not affect viability of HD11 cells.

The effect of IMMP on the viability of HD11 was determined using the Alamar blue viability assay. The concentrations of IMMP (0.6-2.5 mg/mL) tested for toxicity on HD11 cells did not result in differences in viability as compared to the unstimulated control, while Triton X-100 treated cells completely prevented Alamar blue production, as shown in Fig. 1.

Cell viability of HD11 cells after 24 h stimulation with 0.625 – 2.5 mg/mL IMMP is shown as relative cell-viability compared to only media (Unstimulated:’Unst’). Data are represented as means ± SEM of 3 independent experiments performed in duplicate.

### IMMP stimulation changes morphology of chicken HD11 cells

**Fig. 2:**
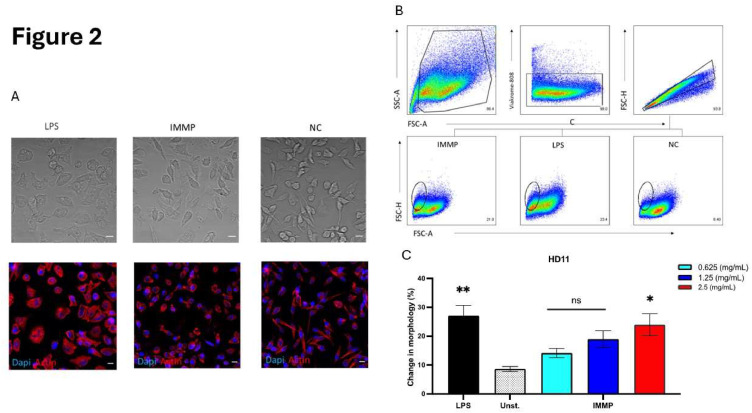
IMMP stimulation induces morphological changes in HD11 cells.

In the next set of experiments the potential of IMMP to activate chicken cells was tested on chicken macrophages and compared with LPS-treated cells. Interestingly, upon 24 h of stimulation with IMMP morphological changes in HD11 cells were observed when compared to unstimulated cells. Brightfield as well as fluorescent confocal images (Fig. 2A) showed that HD11 cells became smaller in size, had less cytoplasmic protrusions, and contained more intracellular vacuoles upon IMMP and LPS stimulation as compared to unstimulated control cells. This observation was confirmed by flow cytometry which showed a shift of the HD11 cell population towards the left in the forward scatter in the FSC-A versus SSC-A dot plots, which means the cells are becoming smaller (Fig. 2B). Quantification of this shift showed that IMMP stimulation significantly affected the size of HD11 cells as compared to unstimulated control cells, as shown in Fig. 2C. This effect was similar to the morphological changes that LPS stimulation induced.

(A) Confocal images (brightfield and fluorescent) of HD11 cells after stimulation with IMMP (2.5 mg/mL), LPS (100 ng/mL), or media only (NC). (B) Dot plots showing changes in the scatter plot of HD11 after stimulation with IMMP (0-2.5 mg/mL), LPS (100 ng/mL). Circled, at a low FSC-H level, is the new distinct population of HD11 cells (C) Graph showing the percentage of HD11 cells with changed morphology after stimulation with IMMP/LPS that fall in the gate shown in Fig. 2B. Data are represented as means ± SEM of 3 independent experiments. Significance compared to the unstimulated control was declared when P < 0.05 (*). Images were taken using a 40x objective lens. scale bar = 10 µm.

### IMMP induced NO production by HD11 cells and chicken MDMs

**Fig. 3:**
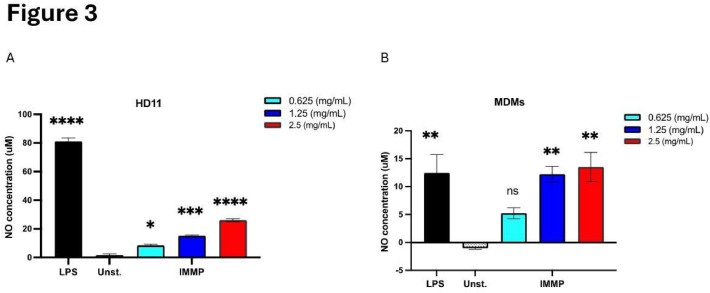
IMMP stimulates nitric oxide production in HD11 macrophages and chicken MDMs.

Besides morphological changes, activation of macrophages can lead to induced nitric oxide production. Stimulation of HD11 cells and chicken MDMs for 24 h with IMMP indeed significantly induced NO production in these cells in a dose dependent manner as compared to the unstimulated control (Fig. 3). Similarly, LPS stimulation also induced NO production by HD11 cells and chicken MDMs. In chicken monocyte derived macrophages, NO production by IMMP (2.5 mg/mL) was substantial but slightly less than the NO produced by 100 ng/ml LPS. In HD11 macrophages, cells that can produce higher absolute concentrations of NO compared to chicken MDMs, IMMP also increased NO production, but to a lower extent than LPS. Interestingly, incubation of HD11 cells with IMMP slightly increased iNOS gene expression, but TLR4 levels were not significantly altered (Supplementary Fig. S2)

(A) NO production by HD11 macrophages after stimulation with IMMP (0-2.5 mg/mL) or LPS (100 ng/mL) for 24h, (B) NO production by chicken MDMs after stimulation with IMMP (0-2.5 mg/mL) or LPS (100 ng/mL) for 24 h. Data are represented as means ± SEM of 3 independent experiments. Significance compared to the unstimulated control was declared when P < 0.05 (*).

### Extracellular TLR4 blocking agent LPS-RS reduces IMMP induced NO production by HD11 cells

**Fig. 4:**
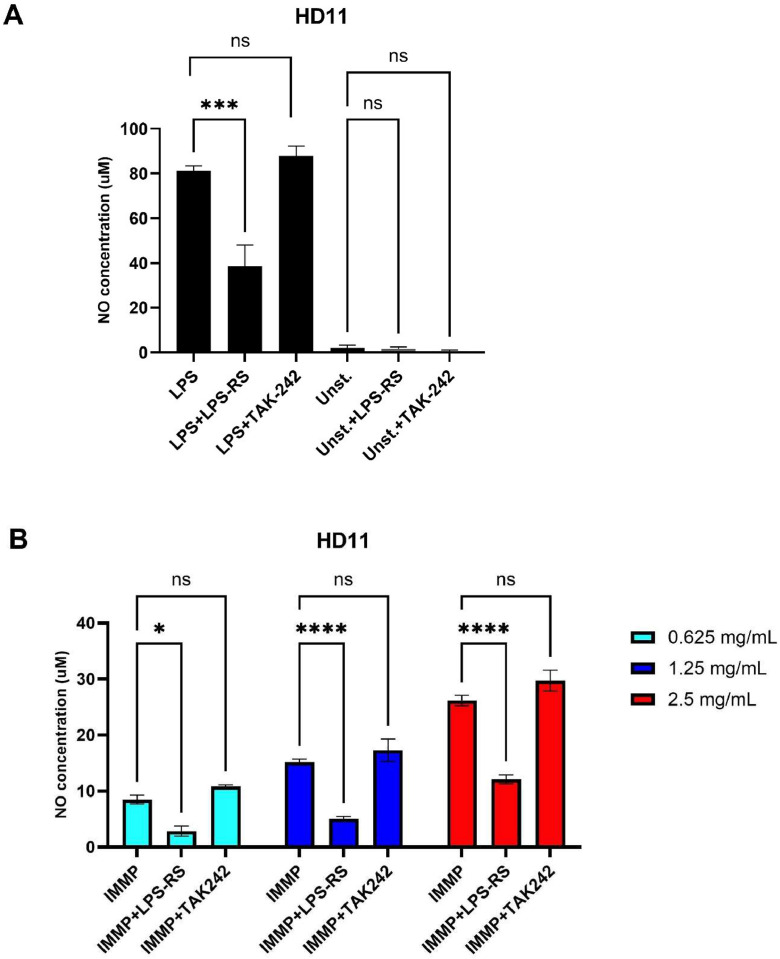
IMMP activates chicken macrophages through TLR4.

To determine if NO production in HD11 cells induced by IMMP was mediated through chicken TLR4, the TLR4 blockers LPS-RS and TAK-242 were used. As shown in Fig. 4A stimulation with LPS in the presence of the extracellular TLR4 blocker LPS-RS resulted in a significant reduction in NO production by HD11 cells, but TAK-242 had no inhibiting effect of LPS-induced activation of HD11 cells. LPS-RS and TAK-242 themselves, in the absence of LPS, did not affect NO production. Similarly, LPS-RS significantly inhibited IMMP-induced NO production by HD11 cell, while again TAK-242 was inactive. These results clearly indicate that IMMP, similar to LPS, can activate chicken macrophages through TLR4.

(A) NO production by HD11 macrophages after incubation with LPS-RS (200 ng/mL) or TAK-242 (2 µg/mL) for 24 h in the presence and absence of LPS (100 ng/mL). (B) Effect of LPS-RS (200 ng/mL) and TAK-242 (2 µg/mL) on IMMP-induced (24 h) NO production by HD11 cells. Data are represented as means ± SEM of 3 independent experiments.

### IMMP stimulation results in enhanced bead uptake by HD11 cells

**Fig. 5:**
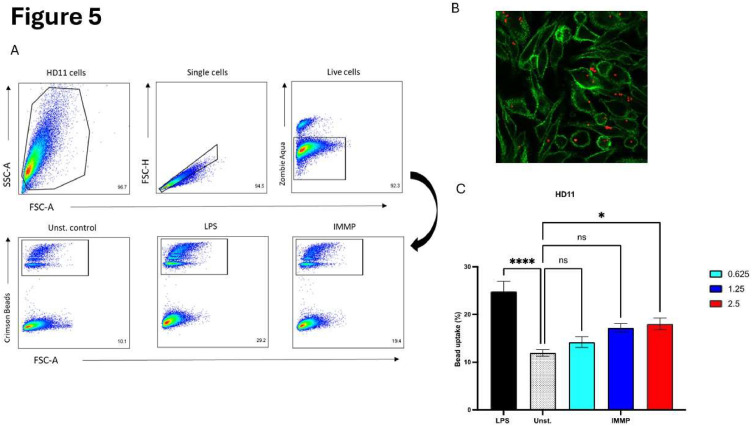
Bead uptake by HD11 cells after stimulation with IMMP.

To determine the effect of IMMP on phagocytosis of macrophages, uptake of crimson beads by HD11 cells was measured by flow cytometry. The gating strategy to determine the bead uptake by HD11 cells after stimulation with IMMP is shown in Fig. 5A. IMMP stimulation significantly increased the phagocytic capacity of HD11 cells as shown by increased bead uptake in a dose dependent manner to a 6 % increase at 2.5 mg/mL IMMP (18% positive cells vs 12 % for the unstimulated control). The effect of IMMP-induced increase in bead uptake by HD11 cells was lower than for LPS treated HD11 cells (13% increase compared to the negative control). Additionally, bead uptake was visualized using confocal microscopy. As shown in Fig. 5B the beads are phagocytosed by the HD11 cells rather than just attached to the surface.

(A) Representative gating strategy to determine bead uptake by HD11 cells after stimulation with IMMP; (B) Confocal image showing bead uptake by HD11 cells. (C) Bead uptake by HD11 cells after stimulation with IMMP (0.6-2.5 mg/mL), LPS (50 ng/mL), and only media for 24 h; Shown are mean +±SEM of 3 independent experiments performed in duplicate.

## Discussion

This study demonstrated that IMMP can activate chicken macrophages through TLR4. Stimulation of HD11 cells as well as chicken MDMs with IMMP resulted in increased nitric oxide production. Activation of HD11 cells by IMMP was confirmed to be TLR4 mediated since co-incubation with the extracellular TLR4 antagonist, LPS-RS, substantially reduced the IMMP induced NO production. In contrast to LPS-RS, NO production of HD11 cells could not be blocked by TAK-242, the intracellular TLR4 inhibitor. This is likely due to the observed difference in the amino acid sequences of the intracellular domain of chicken and human TLR4 (Supplementary Fig. S1). It has been shown that TAK-242 binds to cysteine 747 of the TIR domain in the ICD of human TLR4 (Matsunaga, et al., 2011; Takashima, et al., 2009) and thereby blocks the interaction with TIRAP/MYD88 and TRIF/TRAM, the first adaptor proteins that initiate downstream signaling of the MYD88-dependent and -independent signaling, respectively. Cys747 is present in the chicken sequence but if TAK-242 binds it apparently does not interfere with the interaction with TIRAP/MYD88 (in chicken the TRIF/TRAM pathway is absent in TLR4 signaling (Keestra and van Putten, 2008). The sequence and consequently structural differences in the chicken TIR (and TIRAP/MYD88) proteins compared to human explain the lack of inhibition of chicken TLR4 by TAK-242, but further in-depth structural analysis of the cTLR4-TIRAP/MYD88 may consolidate this finding.

It is interesting to see that IMMP has a similar TLR4-stimulating effect as LPS, the conventional agonist of TLR4. However, the actual binding site of IMMP is different from LPS, based on molecular docking (Silva-Lagos, et al., 2025) and it might also be species dependent since several amino acid residues of human TLR4 involved in binding IMMP are not present in chicken TLR4. This is not surprising considering that LPS binds mainly with the acyl chains of its lipid A domain to the TLR4-MD2 complex, while IMMP only consists of sugar residues and lacks the acyl chain moyeties. However, despite these differences, the functional result of binding IMMP is similar to LPS, at least with respect to the increased production of NO and the increased phagocytosis which has also been described for LPS. The latter result is also in agreement with previous studies describing an increase in phagocytic capacity of HD11 cells after treatment with polysaccharides and glucans (Bar-Dagan, et al., 2023; Han, et al., 2017).

This study specifically investigated the interaction of IMMP with chicken TLR4, mainly based on the observation that the HEK-Blue TLR4 reporter cell line showed a more robust response to IMMP stimulation compared to the HEK-Blue-TLR2 reporter. However, a role for chicken TLR2 cannot be ruled out. Silva-Lagos *et al*. demonstrated that in human THP-1 cells IMMP exhibits immunostimulatory activity through both TLR2 and TLR4 (Silva-Lagos, et al., 2025) and this may also be the case in chicken macrophages. The structural complexity of chicken TLR2, due to its requirement for heterodimerization with either TLR1 or TLR6 poses challenges for clearly delineating its ligand interactions. In addition the use of TLR2-specific inhibitors in chickens is also limited, making it difficult to definitively assess IMMP-TLR2 interactions. Therefore, this study focused exclusively on elucidating the role of chicken TLR4 in IMMP recognition but does not rule out interactions of IMMP with other receptors.

The current study indicated that IMMP can interact directly with chicken macrophages and activate these cells. When considering IMMP supplementation as a potential feed additive in poultry, it can be suggested that IMMP can pass through the gut barrier and interact with the macrophages that are predominantly present in the lamina propria in the chicken gut, separated from the lumen where feed is initially present. An *in vitro* study using a human transwell CaCo-2 cell line model has provided evidence of transfer of prebiotic oligosaccharides through the gut epithelium (Eiwegger, et al., 2010). However, whether similar transport occurs in the avian gut remains unclear and is key to understand if translation of these *in vitro* data to an *in vivo* situation is possible. To address this knowledge gap, further *in vitro* and *in vivo* investigations are warranted, such as employing transwell systems with fluorescently labeled IMMP to assess the extent to which these compounds can directly interact with macrophages in the chicken gut and contribute to intestinal immune function and overall gut health.

Overall, this study demonstrates for the first time that IMMP exerts immunomodulatory effects *in vitro* through TLR4-mediated activation of chicken macrophages. These findings provide a scientific basis for further investigation of IMMP to explore its potential *in vivo* as an immunomodulatory feed additive for poultry production.

## Disclosures

The authors declare no conflict of interest.

## CRediT authorship contribution statement

**Adil Ijaz:** Writing – original draft, Methodology, Formal analysis, Data curation. **Max Putman:** Methodology. **Femke Broere:** Writing – review & editing, Supervision. **Victor P.M.G. Rutten:** Writing – review & editing. **Christine A. Jansen:** Writing – review & editing, Supervision. **Edwin J.A. Veldhuizen:** Writing – review & editing, Supervision, Formal analysis, Conceptualization.

## Appendix Supplementary materials

Image, application 1

## Footnotes

Supplementary material associated with this article can be found, in the online version, at doi:10.1016/j.psj.2025.105690.
