# Supplementary material for: Activation of chicken macrophages by isomalto/malto-polysaccharide (IMMP) is facilitated by toll-like receptor 4 (TLR4)
Source: Poult Sci. 2025 Aug 14;104(11):105690. doi: 10.1016/j.psj.2025.105690 (PMC12397863; doi:10.1016/j.psj.2025.105690)
Supplement: Supplementary file 1 [file mmc1.docx]

**Supplementary data, Ijaz et al.**

Human ----MMSASRLAGTLIPAMAFLSCVRPESWEPCVEVVPNITYQCMELNFYKIPDNLPFST

Mouse -----MMPPWLLARTLIMALFFSCLTPGSLNPCIEVVPNITYQCMDQKLSKVPDDIPSST

Chicken MPSRAAPTALTLGVLLQLLLVLSLLAGCIPSPCLEVIPSTAFRCTGQNISGVPAEIPNTT

.. . : .:* : .**:**:*. :::* :: :* ::* :*

Human KNLDLSFNPLRHLGSYSFFSFPELQVLDLSRCEIQTIEDGAYQSLSHLSTLILTGNPIQS

Mouse KNIDLSFNPLKILKSYSFSNFSELQWLDLSRCEIETIEDKAWHGLHHLSNLILTGNPIQS

Chicken LDLDLSFNSLKLLSSNYFSSVPELQFLDLSRCHIHTIEDNSFVDLYNLSTLILTANSLQH

::*****.*: * * * ...*** ******.*.**** :: .* :**.****.*.:*

Human LALGAFSGLSSLQKLVAVETNLASLENFPIGHLKTLKELNVAHNLIQSFKLPEYFSNLTN

Mouse FSPGSFSGLTSLENLVAVETKLASLESFPIGQLITLKKLNVAHNFIHSCKLPAYFSNLTN

Chicken LGLAAFHGLTSLKKLVLVETSISSLSDLPIGHLNTLQELNLGHNNIASLKLPKYFANLTS

:. .:* **:**::** ***.::**..:***:* **::**:.** * * *** **:***.

Human LEHLDLSSNKIQSIYCTDLRVLHQMPLLNLSLDLSLNPMNFIQPGAFKEIRLHKLTLRNN

Mouse LVHVDLSYNYIQTITVNDLQFLRENPQVNLSLDMSLNPIDFIQDQAFQGIKLHELTLRGN

Chicken LRHLSFSSNNITYISKGDLDALRETNRLNLTLVLSLNNIKYIQSGSFAKIHLGELILRSS

* *:.:* * * * ** *:: :**:* :*** :.:** :* *:* :* **..

Human FDSLNVMKTCIQGLAGLEVHRLVLGEFRNEGNLEKFDKSALEGLCNLTIEEFRLAYLDYY

Mouse FNSSNIMKTCLQNLAGLHVHRLILGEFKDERNLEIFEPSIMEGLCDVTIDEFRLTYTNDF

Chicken FENLNAMHSSLQGLAGLQVNRLIVGEFTNILKITAFQNGLLSGLCQVQMQEFVLMCFREF

*:. * *::.:*.****.*:**::*** : :: *: . :.***:: ::** * :

Human LDDIIDLFNCLTNVSSFSLVSVTIERVKDFSYNFGWQHLELVNCKFGQFPTLKLKSLKRL

Mouse SDDIVKFH-CLANVSAMSLAGVSIKYLEDVPKHFKWQSLSIIRCQLKQFPTLDLPFLKSL

Chicken ENDTDTLFDCIGNVTTIRLVDLNLETLSEVPMFSQVKHLEWKRCKFQELPAEKLSLFKEL

:* :. *: **::: *..:.:: :.:.. : *. .*:: ::*: .* :* *

Human TFTSNK-----GGNAFSEVDLPSLEFLDLSRNGLSFKGCCSQSDFGTTSLKYLDLSFNGV

Mouse TLTMNK-----GSISFKKVALPSLSYLDLSRNALSFSGCCSYSDLGTNSLRHLDLSFNGA

Chicken RVLRITKSKDLNGFEQKFGSLTHLEVVDLSENRLSFLTCCSPKFPRSPNLKHLNLSFNSD

. . .. . *. *. :***.* *** *** . : .*::*:****.

Human ITMSSNFLGLEQLEHLDFQHSNLKQMSEFSVFLSLRNLIYLDISHTHTRVAFNGIFNGLS

Mouse IIMSANFMGLEELQHLDFQHSTLKRVTEFSAFLSLEKLLYLDISYTNTKIDFDGIFLGLT

Chicken ISLTGEFANLRNLLYLDLQHTKLIHHGTYPVFLLLQKLIYLDISYTKTHVMSHLIFHGLN

* ::.:* .*.:* :**:**:.* : :..** *.:*:*****:*:*:: . ** **.

Human SLEVLKMAGNSFQENFLPDIFTELRNLTFLDLSQCQLEQLSPTAFNSLSSLQVLNMSHNN

Mouse SLNTLKMAGNSFKDNTLSNVFANTTNLTFLDLSKCQLEQISWGVFDTLHRLQLLNMSHNN

Chicken SLQVLKMAGNSFENNTLTNNFENVRRLRILDISSCKLVWVDQSTFNALSELKELIISNNK

**:.********::* *.: * : .* :**:*.*:* :. .*::* *: * :*:*:

Human FFSLDTFPYKCLNSLQVLDYSLNHIMTSKKQELQHFPSSLAFLNLTQNDFACTCEHQSFL

Mouse LLFLDSSHYNQLYSLSTLDCSFNRIETSKG-ILQHFPKSLAFFNLTNNSVACICEHQKFL

Chicken LLTFDPVTYKPLQALTALDFSNNQMSFLSDSALEILPDSLVLLDISHNLFECSCTHLNFL

:: :*. *: * :* .** * *:: . *: :*.**.::::::* . * * * .**

Human QWIKDQRQLLVEVERMECATPSDKQGMPVLSLN-ITCQMNK**TIIGVSVLSVLVVS****VVAVL**

Mouse QWVKEQKQFLVNVEQMTCATPVEMNTSLVLDFNNSTCYMYKTIISVSVVSVIVVSTVAFL

Chicken KWVKEKQDLLRNKHSMICHTPAYMKNMSLSNFDMSSCHPNPTTVACSVTVLLAAGVFLFL

:*:*:::::* : . * * ** : : .:: :* * :. ** ::..... .*

Human VYKFYFHL----MLLAGCIKYGRGENI**YDAFVIYSSQDEDWVRNELVKNLEEGVPPFQLC**

Mouse IYHFYFHL----ILIAGCKKYSRGESIYDAFVIYSSQNEDWVRNELVKNLEEGVPRFHLC

Chicken IYKYYFQLYYSLVLLSGCKHSAERGDIYDAFVIHSSKDQEWVMKELVEPLEEGKPPFQLC

:*::**:* :*::** : .. .*******:**::::** :***: **** * *:**

Human **LHYRDFIPGVAIAANIIHEGFHKSRKVIVVVSQHFIQSRWCIFEYEIAQTWQFLSSRAGI**

Mouse LHYRDFIPGVAIAANIIQEGFHKSRKVIVVVSRHFIQSRW**C**IFEYEIAQTWQFLSSRSGI

Chicken LYFRDFLPGVPIVTNIIQEGFLSSRNVIAVISADFLESKW**C**SFEFDIARSWQLVEGKAGI

*::***:***.*.:***:*** .**:**.*:* .*::*:** **::**::**::..::**

Human **IFIVLQKVEKTLLRQQVELYRLLSRNTYLEWEDSVLGRHIFWRRLRKAL**LDGKSWNPEGT

Mouse IFIVLEKVEKSLLRQQVELYRLLSRNTYLEWEDNPLGRHIFWRRLKNALLDGKASNPEQT

Chicken IMIILGEVDKTLLRQRLGLSRYLRRNTYLEWKNKEISRHIFWRQLTSVLLEGKKWNHEEI

*:*:* :*:*:****:: * * * *******::. :.******:* ..**:** * *

Human VGTGCNWQEATSI

Mouse AEEEQETATWT--

Chicken KLM----------

**B**

| **Domain** | **human vs chicken** | **human vs mouse** | **chicken vs mouse** |
| --- | --- | --- | --- |
| **TLR4** | **46%** | **67%** | **44%** |
| **ECD** | **32%** | **62%** | **33%** |
| **ICD** | **57%** | **87%** | **53%** |
| **TIR** | **60%** | **92%** | **57%** |

***Supplementary Fig. S1****. A: Sequence alignment and Identity matrix of human, mouse, chicken TLR4 protein. Asterisk (*) shows fully conserved residues, colon (:) shows conservation between groups of strongly similar properties, dot (.) indicates conservation of groups of weakly similar properties. Cysteine residue 747 of the human TLR4 sequence, and its homologue residue in mouse and chicken TLR4, which is the binding target of TAK-242 is indicated in red/bolt. The extracellular domain of hTLR4 is highlighted in yellow, the transmembrane domain in green, the intracellular domain in light blue which contains the TIR domain (Grey). B: matrix indicating sequence homology of human, mouse and chicken TLR4 and its domains.*

*
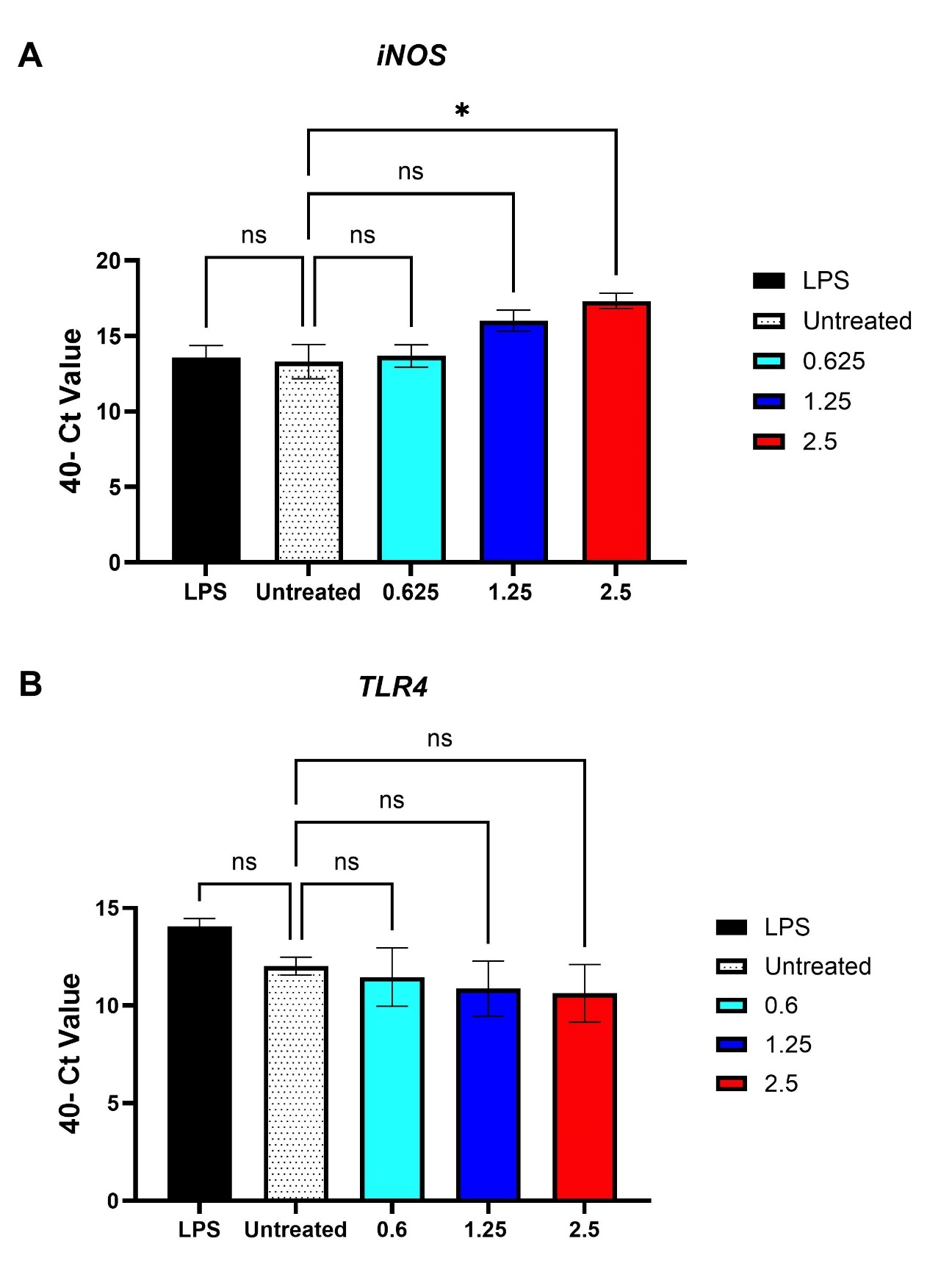
*

***Supplementary Fig. S2.IMMP Induced gene expression of iNOS but not TLR4.***

*HD11 cells were incubated with 100 ng/mL LPS of IMMP (0-2.5 mg/mL) after which mRNA was extracted using Trizol. Gene expression of iNOS and TLR4 were determined using GAPDH as reference gene. (A) iNOS , (B) TLR4.Shown are average + St.Dev of 3 independent experiments performed in duplicate.*
